# A Pilot Study to Non-Invasively Track PIK3CA Mutation in Head and Neck Cancer

**DOI:** 10.3390/diagnostics8040079

**Published:** 2018-11-29

**Authors:** Henri Schmidt, Arutha Kulasinghe, Richard J.N. Allcock, Lit Yeen Tan, Elisa Mokany, Liz Kenny, Chamindie Punyadeera

**Affiliations:** 1 The School of Biomedical Sciences, Institute of Health and Biomedical Innovation, Queensland University of Technology, Kelvin Grove 4059, Queensland, Australia; henri.schmidt@connect.qut.edu.au (H.S.); arutha.kulasinghe@qut.edu.au (A.K.); 2 Translational Research Institute, Queensland University of Technology, Woolloongabba 4102, Queensland, Australia; 3 School of Biomedical sciences, The University of Western Australia, Nedlands 6009, Western Australia, Australia; richard.allcock@uwa.edu.au; 4 Pathwest Laboratory Medicine WA, Nedlands 6009, Western Australia, Australia; 5 SpeeDx Pty. Ltd., National Innovation Centre, Australian Technology Park, Eveleigh Sydney 2015, New South Wales, Australia; littyt@speedx.com.au (L.Y.T.); elisam@speedx.com.au (E.M.); 6 Central Integrated Regional Cancer Service, Royal Brisbane and Women’s Hospital, Herston 4029, Queensland, Australia; lizkenny@bigpond.net.au

**Keywords:** circulating tumour (ctDNA), liquid biopsies, head and neck squamous cell carcinoma, head and neck cancers, diagnosis, monitoring

## Abstract

Background: PIK3CA pathways are the most frequently mutated oncogenic pathway in head and neck squamous cell carcinoma (HNSCC), including virally driven HNCs. PIK3CA is involved in the PI3K-PTEN-mTOR signalling pathway. PIK3CA has been implicated in HNSCC progression and PIK3CA mutations may serve as predictive biomarkers for therapy selection. Circulating tumour DNA (ctDNA) derived from necrotic and apoptotic tumour cells are thought to harbour tumour-specific genetic alterations. As such, the detection of PIK3CA alterations detected by ctDNA holds promise as a potential biomarker in HNSCC. Methods: Blood samples from treatment naïve HNSCC patients (*n* = 29) were interrogated for a commonly mutated PIK3CA hotspot mutation using low cost allele-specific Plex-PCR^TM^ technology. Results: In this pilot, cross sectional study, PIK3CA E545K mutation was detected in the plasma samples of 9/29 HNSCC patients using the Plex-PCR^TM^ technology. Conclusion: The results of this pilot study support the notion of using allele-specific technologies for cost-effective testing of ctDNA, and further assert the potential utility of ctDNA in HNSCC.

## 1. Introduction

Head and neck squamous cell carcinomas (HNSCCs) are the 6th most common cancer globally and arise from multiple anatomical sites [1]. Known risk factors for HNSCCs include tobacco exposure, alcohol consumption, betel nut chewing and infection with oncogenic viruses, such as Epstein-Barr virus (EBV) and Human papillomavirus (HPV, in particular HPV 16–18) [1]. Global incidence of HNSCCs is 500,000 new cases annually with 350,000 associated deaths [1].

Current diagnostic methods rely on conventional medical imaging (MRI/CT/PET), clinical assessment and histological (p16^INK4a^) staining of tissue biopsies to determine the HPV status. Intra-tumour heterogeneity, an added layer of complexity, may hinder treatment strategies that are based on a single tumour-biopsy sample. Differences in somatic events spatially within the primary tumour, between primary and metastatic sites, between individual metastatic sites; as well as temporally, within the same single biopsy site, contribute to treatment failure and drug resistance [2,3]. As such, single-site tumour-biopsy as a means of interrogating the genetic landscape of a tumour is inherently biased, with said biopsies akin to looking through a keyhole of a much larger landscape [4,5]. Numerous studies have shown that when multiple biopsies of the same tumour tissue were analysed, there were vast genetic diversity between samples [2,5,6,7]. ‘Cell free’ circulating DNA (cfDNA) addresses this unmet clinical need, whereby genetic alterations that are representative of a tumour’s genetic profile and clonality are easily accessed via body fluids, in a non-invasive manner. Analysis of cfDNA in particularly, cell-free tumour DNA (ctDNA) presents an avenue for unbiased characterisation of a patient disease status, as well as response to treatment, as the DNA fragments in circulation should represent all phenotypes within a given tumour and may provide a more thorough profile of the tumour heterogeneity in turn facilitating better treatment decisions [8,9,10].

More recently, genetic interrogation of HNSCCs has revealed several common somatic mutations amongst the different tissue subtypes; including aberrations within *TP53*, *CDKN2A*, *PIK3CA*, *NOTCH* and *HRAS* oncogenes [11]. Unfortunately, a readily available genetic target has yet to be found across the different subtypes of HNSCCs [11,12]; further obscuring the lack of clinically available biomarkers for the early evaluation of disease, monitoring of treatment response or risk of relapse [13,14,15,16].

Of note, the phosphatidylinositol-4,5-bisphosphate 3-kinase, catalytic subunit α, *PIK3CA* gene, is frequently reported to harbour helical and kinase domain point mutations [17]. Of note is that *PIK3CA* is also prominent in other solid cancers, including breast cancer [18], prostate cancer [19] and colorectal cancers [20] and has been shown to induce oncogenic transformation in numerous in vivo and in vitro studies [21,22,23]. Hence, *PIK3CA* mutation represents an important somatic event within solid tumours and holds interest as a target for blood-based cancer tests, including detection of HNSCCs.

Characterisation of ctDNA requires highly sensitive assays for the detection of rare mutant molecules amongst the wild-type DNA; platforms include Bead-based emulsion PCR (BEAMing) [24], Ultra-deep [25] and High coverage Next Generation Sequencing (NGS), Digital Droplet PCR (ddPCR) [26,27], Amplification-refractory mutation system (ARMS)-PCR [28], and Mutant-enriched PCR [29]. The high cost of NGS sequencing for the detection of ctDNA has fuelled a push for less-expensive, allele-specific amplification technologies [28] with similar sensitivities and specificity to high-depth high-coverage NGS runs.

PlexPrime™ and PlexZyme™ is an allele-specific amplification technology to detect mutations in a real-time PCR to discriminate rare mutant alleles from ‘wild-type’ backgrounds. The Plex-PCR^TM^ technology utilises 5’ primer annealing with a custom insert, and 3’ locking at the mutation site, producing allele-specific amplicons with a ‘barcode insert’ [30]. Complementary inserts produced during real-time PCR are recognised by specific PlexZymes, allowing for single-nucleotide allele-specific detection.

We hypothesised that mutations in cfDNA can be amplified using the Plex-PCR^TM^ DNA amplification technology. In this pilot study, our aims were to determine whether Plex-PCR^TM^ technology could be used to detect PIK3CA p.E545K mutation in HNSCC samples.

## 2. Materials and Methods

### 2.1. Subjects and Study Design

Ethics approval was obtained from the Metro North and South Health Service District Human Research Ethics Committee in accordance with the National Health and Medical Research Council’s guidelines to collect samples from HNSCC patients attending the Royal Brisbane and Women’s Hospital (RBWH) and Princess Alexandra Hospital (PAH) (HREC/12/QPAH381). All methods were performed in accordance with these ethical guidelines and regulations. The human ethics committee of the Queensland University of Technology has also approved this study (Ethics number: 1400000617). Patients were eligible to take part in this study if they had primary or metastatic HNSCC, with no prior treatment. Written informed consent was obtained from participants and 10 mL blood samples were obtained from patients presenting to the clinic. A pathology report was included for each patient, which contained pathological staging of the tumour, histopathological classification and HPV status based on p16^INK4A^ immunohistochemistry (IHC) (Table 1).

### 2.2. Isolation and Quantification of DNA from Blood Samples

Peripheral blood was drawn into EDTA tubes. Within two hours of collection, tubes were subjected to centrifugation at 800 × g for 10 min at room temperature. 1 mL aliquots of plasma were transferred to 1.5 mL tubes and centrifuged again at 16,000 × g for 10 min to pellet remaining cellular debris. Plasma supernatants were transferred to fresh tubes and stored at −80 °C. cfDNA was isolated from plasma using the QIAamp Circulating nucleic acid kit (Qiagen, Hilden, Germany) according to manufacturer’s instructions. cfDNA amounts were quantified using the Qubit 3.0 Fluorometer (ThermoFisher Scientific, Waltham, MA, USA), and re-queried on the Agilent 2100 Bioanalyser (Agilent, Santa Clara, CA, USA) using a high sensitivity DNA kit (Agilent Technologies, Santa Clara, CA, USA). The concentrations of cfDNA are shown in Table 1.

### 2.3. Allele-Specific PlexPrime/PlexZyme Real-Time PCR

Given the increasing interest in PIK3CA’s oncogenic transformative effects, the helical domain mutation, p.E545K, was chosen as a suitable candidate for HNSCC ctDNA plasma testing as this point mutation has been widely documented with a high frequency in HNSCC tumour tissues (11–33% of HNC) [31]. Allele-specific detection of PIK3CA p.E545K was achieved using SpeeDx PlexPrime and PlexZyme technology in real-time PCR as previously published [32]. Breast cancer cell line, MCF7 (ATCC^®^HTB-22), was used as a p.E545K positive control, with HNSCC cell line FaDu (ATCC^®^HTB-43) used as a ‘wild-type’ negative control. Genomic DNA was harvested from MCF7 and FaDu cell lines and used in serial dilution, for testing and optimisation of the PlexPrime/PlexZyme assay. Allele-specific assays containing 1 mM of PlexZyme probe mastermix, 2 mM MgCl_2_, 1 mM of E545K PlexPrime mix, in a final reaction volume of 15 µL in 384-well plates. These reactions were thermocycled according to the manufacturer’s instructions (SpeeDx, Sydney, Australia); 95 °C for 2 min followed by 10 cycles of 95 °C for 5 s, 61 °C for 30 s (−0.5 °C/cycle), and 40 cycles of 95°C for 3 s, 52 °C for 60 s. All reactions were cycled on the QuantStudio 7 Flex Real-Time PCR system (Applied Biosystems, Waltham, MA, USA).

### 2.4. Wild-Type qPCR

PowerUp SYBR Green Mastermix (ThermoFisher Scientific, Waltham, MA, USA) was used for amplification of PIK3CA p.E545 regions. Briefly, reaction mixtures contained 5 µL of SYBR Green master mix (ThermoFisher Scientific, Waltham, MA, USA), 3 µL of DNA/RNase-free water, and 1 µL of forward and reverse primers; template DNA (2 ng) or no-DNA was added to each reaction in duplicate, for a final reaction volume of 11 µL in 384-well plates. These reactions were thermocycled using standard SYBR Green cycling conditions on the QuantStudio 7 Flex Real-Time PCR system (Applied Biosystems, Waltham, MA, USA). Patient sample reactions contained 2 ng of ctDNA template (or no-DNA), run in duplicate for both SpeeDx allele-specific amplification and SYBR wild-type amplification. MCF7 and FaDu Cell-lines served as positive and negative controls for all runs. E545K mutations were called based on delta Ct (∆CT) values observed between assays.

### 2.5. Mutation Calling

Mutations were called based on the delta Ct method (Mutation assay Ct—Wild-type assay Ct). MCF7 E545K Ct values were subtracted from MCF7 E545 WT Ct values, to determine the cut-off for ‘pure’ mutation; FaDu delta Ct values served as the threshold for ‘pure’ wild-type. Samples found to be significantly above the wild-type threshold value were deemed E545K positive, with samples at or below threshold deemed E545K negative.

## 3. Results

The clinicopathological features of the HNSCC patients investigated are shown in Table 1. HNSCC disease was classified using the American Joint Committee on Cancer (AJCC) ‘Tumour, Node, and Metastasis’ (TNM) staging system. Patients enrolled in the study presented with stage III–IV, treatment naïve HNSCC. None of the patients had evidence of distant metastatic disease upon diagnosis.

### E545K Allele-Specific Screening

**Figure 1:**
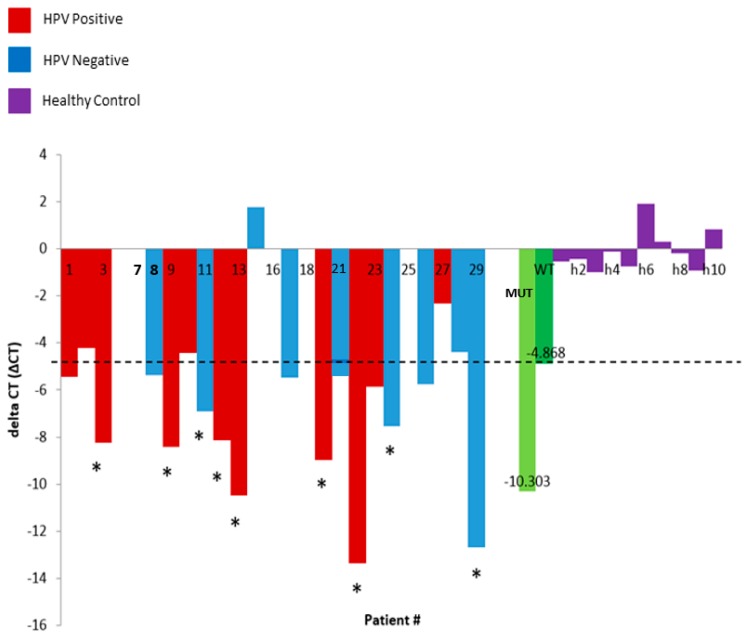
The bar graph showing the delta Ct (∆CT) of HPV Positive (Red) and HPV Negative (Blue) plasma samples, w/high frequency of mutation in the positive control (MUT—light green) and a low mutation call in a wild type negative control (WT—dark green), and healthy control samples (purple: h1–h10). Samples found above the WT ∆CT (represented by the dotted line) are positive for the E545K somatic mutation. Asterisk (*) indicates samples which are above the ∆CT threshold, hence will likely harbour the E545K somatic mutation; samples at and/or below the threshold are less likely to be positive. Patient # correspond to the individual patient numbers.

PIK3CA p.E545K is a frequent somatic mutation in HNSCC tumours [31]. The PIK3CA E545K assay (SpeeDx) confirmed the presence of this mutation in an orthogonal test. We then extracted cfDNA from an independent cohort of HNSCC patients (*n* = 29), and tested for the presence of the p.E545K using this technique; 9/29 plasma samples were positive for the p.E545K somatic mutation, with ∆CT values above the ‘wild-type’ (WT) threshold (Figure 1). ∆CT of cfDNA from healthy control plasma samples was found to be consistently below the threshold, highlighting the specificity of the assay (Figure 1).

## 4. Discussion

A liquid biopsy for the assessment of ctDNA offers an effective means of assessing tumour changes compared to standard tissue biopsy. Liquid biopsies overcome the challenges involved in obtaining repeat tumour biopsies over the course of treatment and can represent a more global view of the heterogeneity present in the tumour. Moreover, ctDNA analysis can be conducted in real-time and offers highly sensitive and specific assays.

ctDNA has previously been explored in other solid tumours, but there are fewer studies pertaining to HNSCCs [33,34,35]. ctDNA is frequently reported as a predictor of treatment resistance and/or failure in breast [36], prostate [37], lung [38], melanoma [27], and colorectal cancers [34]; however, the potential clinical utility of ctDNA as diagnostic, prognostic and/or predictive biomarkers in HNSCCs has not thoroughly been explored [39]. This may partially be attributed to the inability to amplify and detect particular mutations in ctDNA, due to the low copy number. In this pilot cross sectional study, we used commonly mutated PIK3CA E545K mutation to demonstrate a novel PCR technology, PlexPrimer/PlexZyme. Using the sensitive allele-specific PlexPrimer/PlexZyme technology from SpeeDx™, we were able to detect this mutation in 9/29 stage 3–4 HNSCC patient blood samples, demonstrating the feasibility of an allele-specific test for ctDNA testing.

Previous studies have investigated the ctDNA as a potential diagnostic/prognostic biomarker in HNSCCs, albeit in small patient cohorts (*n* = 3 and *n* = 10, respectively) [33,34]. In 2015, Wang et al. demonstrated that ctDNA could be detected in the plasma and saliva of HNSCC patients [35]. Highlighted in the Wang et al. study, ctDNA was more readily detected in the saliva of oral cavity cancer patients, with other cancer sites (oropharynx, larynx and hypopharynx) more effectively sampled by plasma ctDNA. This is speculated to be a result of the tumour’s anatomical location and directional shedding of ctDNA into local body fluids/surrounding vasculature. However, Wang et al. study relied heavily on massively parallel ‘deep’ sequencing methods for the detection of very-low levels of somatic mutations and HPV-16 DNA in the ctDNA. The high-cost of these methods makes them impractical for largescale screenings of ‘at-risk’ individuals, as well as monitoring of patients (requiring the testing of serial samples over a course of treatment). ‘Time-to-result’ for NGS runs must also be accounted for when using ‘deep’ sequencing to query plasma and saliva for minute fractions of ctDNA. More recently, studies have shown that high NGS coverage depth competes with sequencing errors to balance mutation identification with low false discovery rate [10,40], Shu et al. 2017 showed that a 300× mean coverage is the minimum requirement for mutation detection at a mutation allele frequency (MAF) cutoff of 1%. Shu et al. also highlighted that increasing the coverage depth only slightly increased mutation detection in the ctDNA [10]. The increased potential for false discovery/errors of single nucleotide variants (SNVs) in high depth sequencing, particularly in plasma sequencing, where ctDNA fractions can exist at 0.01% of total cell-free DNA, may impact downstream analysis and clinical action based on NGS data.

For the above discussed reasons, the use of allele-specific amplification techniques has emerged as an alternative to NGS in the search for common cancer hotspot SNVs; particularly for its ease-of-use in clinical workflows. Through the use of PIK3CA E545K Plex-PCR^TM^ assay, we have demonstrated the detection of a common hotspot mutation (p.E545K) in the plasma of 9/29 HNSCC patients. *PIK3CA* is a prominent oncogene, reported as the most commonly aberrant gene within solid tumours; the p110α catalytic subunit is frequently identified and implicated in oncogenesis of many solid tumours, as well as in HNSCCs tumours [41]; with common ‘hotspot’ mutations within its helical domain (p.E545K) and kinase domain (p.H1047L/R) shown to cause oncogenic transformation in mouse models [42]. Interestingly, sequencing studies show approximately 21% of HNSCCs contain PIK3CA amplifications and/or somatic mutations, particularly HPV positive HNSCCs [11]. This trend can also be seen in our study, with six out of the nine HNSCC patients positive for the E545K mutation, also being HPV positive. This provides early data, which suggests that PIK3CA hotspot mutations, may be a candidate for HNSCC patient screening. However, for clinical implementation, a number of commonly mutated genes would need to be validated as HNSCC do not have exclusive tumour specific mutations.

For HPV-negative tumours, mutations are clustered within the ‘RTK-RAS-PI3K’ pathways and cell cycle regulation (*CCND1*), with common loss/inactivation of *CDKN2A* and *TP53* tumour-suppressors and dysregulation of cell-proliferation and growth [11,43]. Conversely, in HPV-positive tumours activating alterations frequently occur in *PIK3CA*, *FGFR3* and E2F1, with HPV-oncoproteins E6 and E7 causing inactivation of wild-type *TP53* and *RB1* functions in cell cycle control [43,44]. Allele-specific technologies that detect common SNVs in *TP53*, *PIK3CA*, *CDKN2A* and other solid tumour-associated oncogenes may provide alternatives to NGS, allowing for rapid ‘time-to-result’, quick screening, as well as monitoring of ‘at-risk’ patients. For longitudinal evaluation of ctDNA, use of allele-specific technology would allow for ‘personalised’ tracking of mutations of interest, where increases in tumour-specific DNA levels post-treatment might prompt the switching of treatments, prior to the emergence of overt resistant and/or recurrent disease.

## 5. Conclusions

The late-stage clinical presentation of HNSCCs portends to locoregional and/or metastatic disease upon diagnosis, comprising a challenge for clinicians treating HNSCCs; having no clinically available biomarkers for non-invasive, early detection and/or prediction of patient treatment response. Examination of ctDNA in the plasma of HNSCC patients present an opportunity for diagnosis, as well as monitoring of treatment responses, via tracking of tumour-specific genetic alterations. Personalised allele-specific assays developed ‘case-by-case’, would allow for cost-effective monitoring of ctDNA, as well as providing rapid ‘time-to-result’ for a quick clinical turnaround, realising precision-medicine.

## Figures and Tables

**Table 1:** Head and neck cancer patients (*n* = 29) demographics data.

Patient ^#^	Age	HPV Status	TNM Staging	Site	DNA (ng/mL)	Sample Type
1	60–65	Positive	T2N2b	Oropharyngeal	41.6	Plasma
2	55–60	Positive	T2N2bM0	Oropharyngeal	22.5	Plasma
3	55–60	Positive	T3N1	Oropharyngeal	10.2	Plasma
4	65–70	Negative	T3N1	Oropharyngeal	15.4	Plasma
5	60–65	Positive	T3N1a	Oropharyngeal	7.8	Plasma
6	45–50	Positive	T3N2b	Oropharyngeal	10.5	Plasma
7	70–75	Negative	T3N2b	Oropharyngeal	21.8	Plasma
8	55–60	Negative	T3N2b	Oropharyngeal	7.2	Plasma
9	60–65	Positive	T3N2c	Oropharyngeal	38.6	Plasma
10	60–65	Positive	T3N2c	Oropharyngeal	10.2	Plasma
11	65–70	Negative	T3N2c	Oral Cavity	4.5	Plasma
12	60–65	Positive	T3N2c	Oropharyngeal	29.2	Plasma
13	55–60	Positive	T4aN2a	Oropharyngeal	28.4	Plasma
14	60–65	Positive	T4aN2b	Hypopharyngeal	36.4	Plasma
15	60–65	Negative	T4aN2b	Oral Cavity	22.9	Plasma
16	55–60	Negative	T4aN2b	Oral Cavity	4.4	Plasma
17	60–65	Negative	T4aN2b	Oral Cavity	29.7	Plasma
18	60–65	Negative	T4aN2c	Laryngeal	10.3	Plasma
19	65–70	Positive	T4N0	Oral Cavity	21.9	Plasma
20	45–50	Negative	T4N0	Unknown	6.0	Plasma
21	65–70	Negative	T4N0	Oral Cavity	27.1	Plasma
22	65–70	Positive	T4N2b	Oropharyngeal	20.1	Plasma
23	45–50	Positive	T4N2b	Oropharyngeal	32.4	Plasma
24	65–70	Negative	T4N2b	Oral Cavity	9.0	Plasma
25	50–55	Negative	T4N2b	Oropharyngeal	7.7	Plasma
26	35–40	Negative	T4N2b	Oral Cavity	12.0	Plasma
27	Unknown	Positive	T4N2c	Unknown	7.3	Plasma
28	65–70	Negative	T4N0	Oral Cavity	9.8	Plasma
29	60–65	Negative	T4N0	Oral Cavity	8.5	Plasma

# Patient number; HPV: Human papillomavirus; TNM: Tumour-node-metastasis staging.

## References

[1] Marur S., Forastiere A.A. (2016). Head and Neck Squamous Cell Carcinoma: Update on Epidemiology, Diagnosis, and Treatment. Mayo Clin. Proc..

[2] Swanton C. (2012). Intratumor heterogeneity: Evolution through space and time. Cancer Res..

[3] Schmidt H., Kulasinghe A., Kenny L., Punyadeera C. (2016). The development of a liquid biopsy for head and neck cancers. Oral Oncol..

[4] Greaves M., Maley C.C. (2012). Clonal evolution in cancer. Nature.

[5] Gerlinger M., Rowan A.J., Horswell S., Larkin J., Endesfelder D., Gronroos E., Martinez P., Matthews N., Stewart A., Tarpey P. (2012). Intratumor heterogeneity and branched evolution revealed by multiregion sequencing. N. Engl. J. Med..

[6] Hao J.J., Lin D.C., Dinh H.Q., Mayakonda A., Jiang Y.Y., Chang C., Jiang Y., Lu C.C., Shi Z.Z., Xu X. (2016). Spatial intratumoral heterogeneity and temporal clonal evolution in esophageal squamous cell carcinoma. Nat. Genet..

[7] Parker N.R., Hudson A.L., Khong P., Parkinson J.F., Dwight T., Ikin R.J., Zhu Y., Cheng Z.J., Vafaee F., Chen J. (2016). Intratumoral heterogeneity identified at the epigenetic, genetic and transcriptional level in glioblastoma. Sci. Rep..

[8] Roschewski M., Staudt L.M., Wilson W.H. (2016). Dynamic monitoring of circulating tumor DNA in non-Hodgkin lymphoma. Blood.

[9] Abbosh C., Birkbak N.J., Wilson G.A., Jamal-Hanjani M., Constantin T., Salari R., Le Quesne J., Moore D.A., Veeriah S., Rosenthal R. (2017). Phylogenetic ctDNA analysis depicts early-stage lung cancer evolution. Nature.

[10] Shu Y., Wu X., Tong X., Wang X., Chang Z., Mao Y., Chen X., Sun J., Wang Z., Hong Z. (2017). Circulating Tumor DNA Mutation Profiling by Targeted Next Generation Sequencing Provides Guidance for Personalized Treatments in Multiple Cancer Types. Sci. Rep..

[11] Cancer Genome Atlas Network (2015). Comprehensive genomic characterization of head and neck squamous cell carcinomas. Nature.

[12] Kang H., Kiess A., Chung C.H. (2015). Emerging biomarkers in head and neck cancer in the era of genomics. Nat. Rev. Clin. Oncol..

[13] Dahiya K., Dhankhar R. (2016). Updated overview of current biomarkers in head and neck carcinoma. World J. Methodol..

[14] Gerstner A.O. (2008). Early detection in head and neck cancer - current state and future perspectives. GMS Curr. Top. Otorhinolaryngol. Head Neck Surg..

[15] van Ginkel J.H., Huibers M.M.H., van Es R.J.J., de Bree R., Willems S.M. (2017). Droplet digital PCR for detection and quantification of circulating tumor DNA in plasma of head and neck cancer patients. BMC cancer.

[16] Kulasinghe A., Perry C., Jovanovic L., Nelson C., Punyadeera C. (2014). Circulating Tumour Cells in Metastatic Head and Neck Cancers. Int. J. Cancer.

[17] Chiosea S.I., Grandis J.R., Lui V.W., Diergaarde B., Maxwell J.H., Ferris R.L., Kim S.W., Luvison A., Miller M., Nikiforova M.N. (2013). PIK3CA, HRAS and PTEN in human papillomavirus positive oropharyngeal squamous cell carcinoma. BMC Cancer.

[18] Nakauchi C., Kagara N., Shimazu K., Shimomura A., Naoi Y., Shimoda M., Kim S.J., Noguchi S. (2016). Detection of TP53/PIK3CA Mutations in Cell-Free Plasma DNA From Metastatic Breast Cancer Patients Using Next Generation Sequencing. Clin. Breast Cancer.

[19] Sun X., Huang J., Homma T., Kita D., Klocker H., Schafer G., Boyle P., Ohgaki H. (2009). Genetic alterations in the PI3K pathway in prostate cancer. Anticancer Res..

[20] Chang P.Y., Chen J.S., Chang S.C., Wang M.C., Chang N.C., Wen Y.H., Tsai W.S., Liu W.H., Liu H.L., Lu J.J. (2017). Acquired somatic TP53 or PIK3CA mutations are potential predictors of when polyps evolve into colorectal cancer. Oncotarget.

[21] Lau C.E., Tredwell G.D., Ellis J.K., Lam E.W., Keun H.C. (2017). Metabolomic characterisation of the effects of oncogenic PIK3CA transformation in a breast epithelial cell line. Sci. Rep..

[22] Green S., Trejo C.L., McMahon M. (2015). PIK3CA(H1047R) Accelerates and Enhances KRAS(G12D)-Driven Lung Tumorigenesis. Cancer Res..

[23] Van Keymeulen A., Lee M.Y., Ousset M., Brohee S., Rorive S., Giraddi R.R., Wuidart A., Bouvencourt G., Dubois C., Salmon I. (2015). Reactivation of multipotency by oncogenic PIK3CA induces breast tumour heterogeneity. Nature.

[24] Diehl F., Schmidt K., Choti M.A., Romans K., Goodman S., Li M., Thornton K., Agrawal N., Sokoll L., Szabo S.A. (2008). Circulating mutant DNA to assess tumor dynamics. Nat. Med..

[25] Chaudhuri A.A., Lovejoy A.F., Chabon J.J., Newman A., Stehr H., Say C., Aggarwal S., Carter J.N., West R.B., Neal J.W. (2016). CAPP-Seq Circulating Tumor DNA Analysis for Early Detection of Tumor Progression After Definitive Radiation Therapy for Lung Cancer. Int. J. Radiat. Oncol..

[26] Xu Q., Zhu Y., Bai Y., Wei X., Zheng X., Mao M., Zheng G. (2015). Detection of epidermal growth factor receptor mutation in lung cancer by droplet digital polymerase chain reaction. Onco. Targets Ther..

[27] Tsao S.C., Weiss J., Hudson C., Christophi C., Cebon J., Behren A., Dobrovic A. (2015). Monitoring response to therapy in melanoma by quantifying circulating tumour DNA with droplet digital PCR for BRAF and NRAS mutations. Sci. Rep..

[28] Stadler J., Eder J., Pratscher B., Brandt S., Schneller D., Mullegger R., Vogl C., Trautinger F., Brem G., Burgstaller J.P. (2015). SNPase-ARMS qPCR: Ultrasensitive Mutation-Based Detection of Cell-Free Tumor DNA in Melanoma Patients. PLoS ONE.

[29] He C., Liu M., Zhou C., Zhang J., Ouyang M., Zhong N., Xu J. (2009). Detection of epidermal growth factor receptor mutations in plasma by mutant-enriched PCR assay for prediction of the response to gefitinib in patients with non-small-cell lung cancer. Int. J. Cancer.

[30] Tan L.Y., Walker S.M., Lonergan T., Lima N.E., Todd A.V., Mokany E. (2017). Superior Multiplexing Capacity of PlexPrimers Enables Sensitive and Specific Detection of SNPs and Clustered Mutations in qPCR. PLoS ONE.

[31] Giudice F.S., Squarize C.H. (2013). The determinants of head and neck cancer: Unmasking the PI3K pathway mutations. J. Carcinog. Mutagen.

[32] Tabrizi S.N., Tan L.Y., Walker S., Twin J., Poljak M., Bradshaw C.S., Fairley C.K., Bissessor M., Mokany E., Todd A.V. (2016). Multiplex Assay for Simultaneous Detection of Mycoplasma genitalium and Macrolide Resistance Using PlexZyme and PlexPrime Technology. PLoS ONE.

[33] Lebofsky R., Decraene C., Bernard V., Kamal M., Blin A., Leroy Q., Rio Frio T., Pierron G., Callens C., Bieche I. (2015). Circulating tumor DNA as a non-invasive substitute to metastasis biopsy for tumor genotyping and personalized medicine in a prospective trial across all tumor types. Mol. Oncol..

[34] Bettegowda C., Sausen M., Leary R.J., Kinde I., Wang Y., Agrawal N., Bartlett B.R., Wang H., Luber B., Alani R.M. (2014). Detection of circulating tumor DNA in early- and late-stage human malignancies. Sci. Transl. Med..

[35] Wang Y., Springer S., Mulvey C.L., Silliman N., Schaefer J., Sausen M., James N., Rettig E.M., Guo T., Pickering C.R. (2015). Detection of somatic mutations and HPV in the saliva and plasma of patients with head and neck squamous cell carcinomas. Sci. Transl. Med..

[36] Dawson S.J., Rosenfeld N., Caldas C. (2013). Circulating tumor DNA to monitor metastatic breast cancer. N. Engl. J. Med..

[37] Azad A.A., Volik S.V., Wyatt A.W., Haegert A., Le Bihan S., Bell R.H., Anderson S.A., McConeghy B., Shukin R., Bazov J. (2015). Androgen Receptor Gene Aberrations in Circulating Cell-Free DNA: Biomarkers of Therapeutic Resistance in Castration-Resistant Prostate Cancer. Clin. Cancer Res..

[38] Murtaza M., Dawson S.J., Tsui D.W., Gale D., Forshew T., Piskorz A.M., Parkinson C., Chin S.F., Kingsbury Z., Wong A.S. (2013). Non-invasive analysis of acquired resistance to cancer therapy by sequencing of plasma DNA. Nature.

[39] Thomas E., Shaw R.J., Risk J.M. (2005). Monitoring of circulating tumour-associated DNA as a prognostic tool for oral squamous cell carcinoma. Br. J. Cancer.

[40] Sims D., Sudbery I., Ilott N.E., Heger A., Ponting C.P. (2014). Sequencing depth and coverage: Key considerations in genomic analyses. Nat. Rev. Genet..

[41] Samuels Y., Waldman T. (2010). Oncogenic mutations of PIK3CA in human cancers. Curr. Top. Microbiol. Immunol..

[42] Meyer D.S., Koren S., Leroy C., Brinkhaus H., Muller U., Klebba I., Muller M., Cardiff R.D., Bentires-Alj M. (2013). Expression of PIK3CA mutant E545K in the mammary gland induces heterogeneous tumors but is less potent than mutant H1047R. Oncogenesis.

[43] Seiwert T.Y., Zuo Z., Keck M.K., Khattri A., Pedamallu C.S., Stricker T., Brown C., Pugh T.J., Stojanov P., Cho J. (2015). Integrative and comparative genomic analysis of HPV-positive and HPV-negative head and neck squamous cell carcinomas. Clin. Cancer. Res..

[44] Sun W., Califano J.A. (2014). Sequencing the head and neck cancer genome: Implications for therapy. Ann. N. Y. Acad. Sci..

